# WHODrug: A Global, Validated and Updated Dictionary for Medicinal Information

**DOI:** 10.1007/s43441-020-00130-6

**Published:** 2020-02-20

**Authors:** Olof Lagerlund, Sara Strese, Malin Fladvad, Marie Lindquist

**Affiliations:** Uppsala Monitoring Centre, Box 1051, 751 40 Uppsala, Sweden

**Keywords:** Pharmacovigilance, Clinical trial, Patient safety, Adverse event, Standardised Drug Groupings (SDG), Drug coding

## Abstract

The WHODrug medicinal information dictionary is a worldwide source of global medicinal information with the aim to facilitate the coding of medications in clinical trials as well as identification of medication-related problems when monitoring patient safety, thereby supporting the development and usage of effective and safe medications. WHODrug contains individual trade names, active ingredients and additional information such as marketing authorisation holder, country of sale, pharmaceutical form and strength. All related medications are linked using a structured WHODrug alphanumeric code, connecting trade names and variation of the ingredient with the active moiety of the ingredient. Medications in WHODrug are classified using the ATC system and clustered into Standardised Drug Groupings, to allow for grouping of medications with one or more properties in common. The built-in data structure and the classification of medications in WHODrug facilitate various ways of aggregating medications for identification and analysis of possible adverse drug reactions. The different information levels in WHODrug are used to explore the relationship between a medication or a class of medications and an adverse event. By using WHODrug in clinical trials and post-marketing safety, accurate and standardised medication information can be achieved globally and allow easy information exchange. To meet the demands of WHODrug users from the pharmaceutical industry, academia and regulatory authorities, it is relevant to keep the dictionary comprehensive, validated and constantly updated on a global scale.

## What is WHODrug?

WHODrug is a global medicinal information dictionary that contains medicinal products and active ingredients intended for human use, such as active chemical substances, biotherapeutics, vaccines, dietary supplements, herbal remedies, radio pharmaceuticals and diagnostic agents. The information of the medications in WHODrug includes trade name, ingredient, pharmaceutical form, strength, country of sales and marketing authorisation holder (MAH). The development and maintenance of WHODrug is managed by Uppsala Monitoring Centre (UMC), the WHO Collaborating Centre for International Drug Monitoring.

In order to meet the demands of its users and to keep WHODrug current and comprehensive, it is continuously updated and adapted to international standards. All medicinal information is verified in at least one reliable national or international reference or directly from market authorisation holders before inclusion in WHODrug. The active ingredients are defined by globally recognised non-proprietary names and herbal ingredients as the accepted scientific name.

## WHODrug in Practice

WHODrug is used to structure and analyse clinical trial [1] and post-marketing safety [2] data worldwide [3]. The growing global pharmaceutical market and the increased awareness of the importance of patient safety have amplified the importance of a common medicinal database for both drug development and post-marketing safety. The ability to maintain and keep WHODrug quality assured and regularly updated on a global scale has made WHODrug a universal source of medicinal information and it is in 2019 used by over 2300 pharmaceutical and biotech companies, Clinical Research Organisations (CROs), universities and regulatory authorities around the world.

### Clinical Trials

WHODrug is used by pharmaceutical organisations and CROs to allow for efficient analysis of the effect of experimental and concomitant medications when performing clinical trials. Coding medications uniformly is of great importance but can pose a major challenge, especially in multicentric trials, performed on several sites in various countries [1]. Using a standardised and validated dictionary for medicinal information is therefore required.

In the clinical trial, each verbatim from a Case Report Form (CRF) is coded to a corresponding medication in WHODrug. Traditionally, much of the coding has been done manually. However, with the evolvement of technology, the so-called autoencoders have been applied to automate parts of the process. The analysis of data from clinical trials is aided by the fact that all medications in WHODrug are grouped in several different ways, for example by therapeutic indication, pharmacological mechanism or chemical structure. These groupings can be utilised in clinical trial analysis, for example in the specification of exclusion criteria and to identify the frequency of adverse events.

WHODrug is required for reporting of concomitant medications in clinical trial submission by leading regulators in some countries [4, 5] and has evolved to become the most comprehensive and actively used reference dictionary for coding medicinal products in the world.

### Safety Signal Detection

Medicinal products, reported in VigiBase, the WHO global database of individual case safety reports (ICSR), or company post-marketing safety databases, are coded with WHODrug, facilitating interpretation and evaluation of safety signals. WHODrug enables identification and data aggregation at different information levels due to the data structure of the dictionary. This applies both to medications reported as concomitant or interacting, as well as those reported as suspected of having caused an adverse drug reaction (ADR).

ICSRs in VigiBase can be screened to find adverse event signals of previously unrecognised or incompletely documented associations between adverse events and medications. Some examples of the use of WHODrug in safety signal detection include case reports linking a drug to an ADR [6], identification of substandard medications [7], exploring the reporting of adverse events in social media [8, 9], age-related ADRs [10] and country-specific reporting [11].

## The Story of WHODrug

### From Tragedy to Pharmacovigilance

The story of WHODrug starts in 1968 with the formation of the WHO Programme for International Drug Monitoring (PIDM) in the aftermaths of the thalidomide tragedy. In the 1960s, it was discovered that this medication, prescribed for morning-sickness in pregnant women, could cause limb deformities in babies [12]. Because of the thalidomide tragedy, the need for systematic collection of adverse event information both during drug development as well as for already approved medications became apparent. This was the modern starting point of pharmacovigilance (PV), the science and activities relating to the detection, assessment, understanding and prevention of ADRs or any other medication-related problem [13].

As a result of the recognition of the need for international collaboration to avoid future disasters, the WHO PIDM was founded with initially 10 member countries, with existing national systems in place. This network has since then grown to 165 member countries as of 2019. The purpose of the programme is to support member countries in their day to day work with pharmacovigilance and to provide a framework for collaboration on a global scale concerning patient safety issues. One part of the collaboration is that member countries submit ICSRs to the WHO global database, VigiBase, and the collected information is subsequently made available to all member countries for identification and analysis of global and national patient safety issues [14].

In 1978, the Foundation WHO Collaborating Centre for International Drug Monitoring in Uppsala, later known as Uppsala Monitoring Centre (UMC), was founded to support the WHO PIDM, and has since been responsible for managing the technical (WHO ICSR database) and scientific aspects of the WHO worldwide pharmacovigilance network. To enable analysis of the collected information from all member countries in a systematic way, UMC developed a method to structure and standardise medicinal information.

### Development of a Standardised Dictionary for Medicinal Information

When the WHO PIDM was set up, a numerical code uniquely identifying each separate ingredient was created, thereby generating a link between medications with different names in different countries with the same ingredient [15, 16]. This structure laid the foundation for the medicinal dictionary today known as WHODrug Global, and the related WHODrug portfolio of resources.

**Figure 1.**
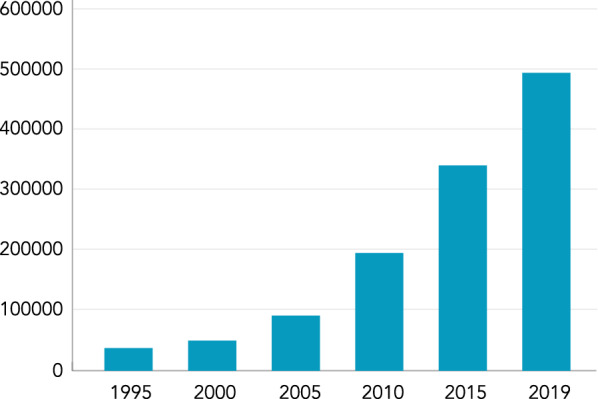
The Number of Unique Medication Names in WHODrug from 1995 to 2019.

From the early 1980s, WHODrug has provided pharmaceutical companies, regulatory authorities and other stakeholders with the opportunity to analyse medicinal data and communicate medicinal information in a standardised way. The dictionary has been produced in different formats over the years, starting as a paper copy, followed by computerised versions on tape and diskettes, and now available as downloadable files and in a web application. Initially most medications in WHODrug originated from the ICSRs submitted to VigiBase through the WHO PIDM. In 1995, the number of medications in WHODrug was 38,700 and it has since increased exponentially, due to proactive inclusion of medicinal products, reaching close to 500,000 unique medication names in 2019 (Fig. 1).

## Data Structure in WHODrug

**Figure 2.**
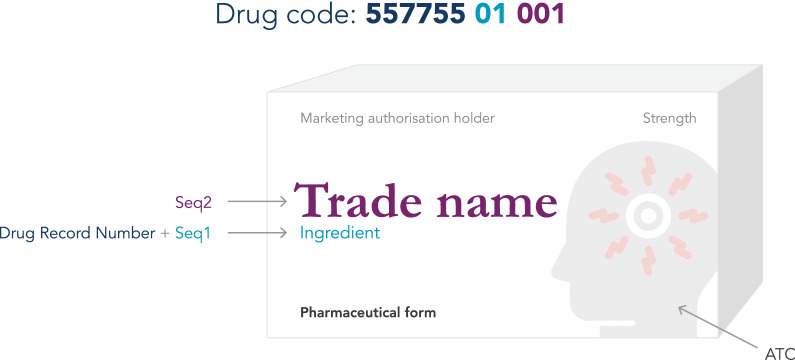
The Drug Code for the Medicinal Product “Trade Name” Consists of the Drug Record Number and Seq 1 (Representing Ingredient) and Seq 2 (Representing Trade Name).

WHODrug consists of medications with individual trade names linked to the active ingredients, pharmaceutical form, strength, MAH and country of sales, as well as a classification according to the WHO Anatomical Therapeutic Chemical classification (ATC) (Fig. 2). WHODrug also contains several umbrella terms for medications not linked to specified active ingredients.

### Link Between Trade Name and Ingredient

All medications in WHODrug are given a unique identifier, a Medicinal Product Identification Number (MPID) to separate them from each other regardless if they differ in ingredient, pharmaceutical form, strength, MAH, country of sales or all the above. To link related medicinal products in a logical way, for example different trade names with the same active ingredient or ingredient combination, WHODrug uses an alphanumeric code, i.e. the Drug code. The Drug code is an 11-character sequence made up of three parts. The first part is 6-characters (Drug Record Number) and represents the ingredients, the second part consists of 2-characters (Seq 1) and represents ingredient variation, and finally the third part consists of 3-characters (Seq 2) representing the name of the medication in WHODrug which can be a trade name, a generic name or an imprecise name (Fig. 2).

### Identifying Non-unique Trade Names

The same trade name is sometimes available with different ingredients and the use of additional information found in WHODrug, such as ingredient, form, strength or country of sales may help to distinguish these non-unique trade names to simplify identification of the correct medical product and to facilitate review of coded data.

### Connecting the Ingredient with the Corresponding Variation(s)

**Figure 3.**
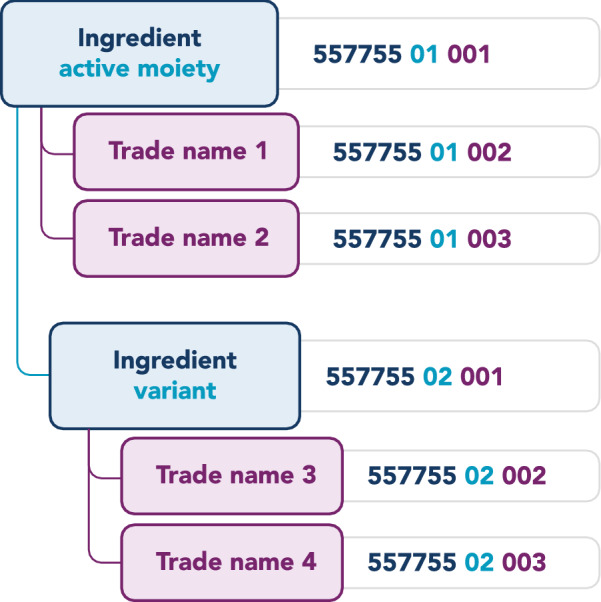
Relationship Between Ingredient, Corresponding Ingredient Variation and Trade Name. Each Medication has a Specific Drug Code Connected by the Same Drug Record Number.

According to the Drug code logic of WHODrug, all ingredient variations, for example salts and plant parts, are connected to the active part of the ingredient, i.e. the active moiety [17]. This means that the Drug Record Number is the same for all ingredient variations with the same active moiety, but the Seq 1 differs. All trade names containing the same ingredient variation are also connected to the same active moiety (Fig. 3).

### Anatomical Therapeutic Chemical Classification in WHODrug

The five-level hierarchical Anatomical Therapeutic Chemical (ATC) classification system is integrated in WHODrug. The ATC system is maintained by the WHO Collaborating Centre for Drug Statistics Methodology [18] and classifies active ingredients according to the organ or system on which they act, as well as their therapeutic, pharmacological and chemical properties. All medications in WHODrug are assigned one or several ATC codes according to the different indications of the medicinal product or ingredients. In addition to the ATC classification system, WHODrug also uses the Herbal ATC (HATC) classification system when classifying herbal remedies. The HATC system was originally developed by UMC to provide a harmonised and global nomenclature for therapeutic classification of herbal remedies.

## Utilise the Structure of WHODrug

**Figure 4.**
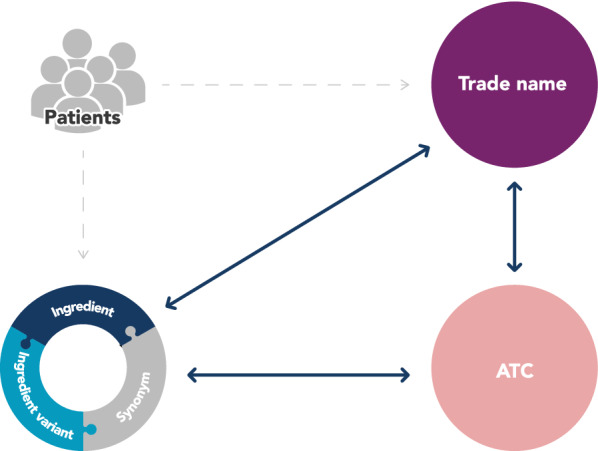
Relation Between Patients, Trade Name, Ingredient and ATC. Note: The reported trade name is always linked to the ingredient of the medicinal product, and hence additional trade names containing the same ingredient can be identified. Each ingredient and trade name is classified with at least one ATC code, providing straightforward identification of other ingredients and trade names within the same ATC classification with similar characteristics.

In addition to the relationship between trade name and ingredient and the connection between the ingredient active moiety and corresponding ingredient variation, the assigned ATC codes in WHODrug make it possible to aggregate the medications in several different ways based on, for example, active ingredients, chemical class or therapeutic use (Fig. 4). These aggregated medications can be used for identification and analysis of adverse events, to identify effects of a certain class of medications in safety signal detection or as a starting point in the development of protocol violation lists and other medication lists of interest in clinical trials [1].

For example, if an adverse event is suspected to be related to a specific medicinal product, by using the built-in structure in WHODrug, all other trade names with the same ingredients can be identified and similar adverse events investigated. In addition, an OTC concomitant medication may have been used to treat the indication of an adverse effect caused by the experimental medication and identification of how frequent that OTC medication is used by the study population will provide more insights into the adverse event itself.

By also looking at the ATC class assigned to the identified medication, the number of medications of interest can be further expanded to find similar medications or other combinations of medications associated with the same adverse event. By analysing all resulting medications, one could identify if the reported adverse event is a class effect, namely a drug effect produced by all members of a pharmaceutically related group of medications. The ATC classification can also be used to identify common indications among the medications taken to look for potential drug–drug interactions.

An example of how the structure of WHODrug can be utilised for signal detection is in the case of desogestrel. The potential relationship between desogestrel and panic attacks was first highlighted as a suspected ADR when identified during signal detection when several reports in VigiBase with the MedDRA term “panic attacks and disorders” were reported together with monotherapy desogestrel. By searching for the ingredient desogestrel in WHODrug, the analysis could be expanded to include all reports with medicinal products containing desogestrel, irrespective of their trade name [19].

## Additional Use of the WHODrug Portfolio

In addition to the WHODrug Global comprehensive medication reference dictionary, the WHODrug portfolio consists of several related resources with the purpose of making the most of WHODrug.

### Standardising Groups of Medications with Shared Properties

The WHODrug Standardised Drug Groupings (SDGs) is an additional WHODrug resource with the purpose of harmonising an unbiased search strategy for the most common groupings of medications based on joint properties that are not easily extracted using the ATC system. The definition of an SDG is a grouping of ingredients having one or more properties in common and can be based on indication, pharmacodynamic or pharmacokinetic properties, chemical structure or any other property of interest. All SDGs allow for modifications by the user, and when altered they are referred to as Customised Drug Groupings (CDGs).

In clinical trials, the SDGs can provide information on how a specific class of drugs may affect the study drug, causing unknown interactions, protocol violations and deviations, and unreported adverse events. Investigators create inclusion/exclusion medication lists as part of the study protocol to monitor medication taken by patients during a trial and SDGs are commonly used to specify these inclusion/exclusion criteria [20].

The SDGs can also be used in various types of patient safety analysis. For example, medications with the same ATC and in the same SDG as nintedanib were investigated to strengthen the original signal of nintedanib-colitis [21].

### Accommodating Regulatory Authority-Specific Demands

Regulatory authorities request certain medication information for the approval and safety surveillance of medications. To aid WHODrug users to accommodate more specific regulatory requirements, WHODrug is continuously adapted to adhere to international standardisation initiatives. Two additional tools are offered, the WHODrug Cross Reference Tool Japan (CRT Japan) and the WHODrug Cross Reference ATC 5 (CR ATC 5). Both these tools use the information in WHODrug and convert it to the format requested by the authorities.

The Japanese Pharmaceuticals and Medical Devices Agency (PMDA) requires WHODrug for submission of concomitant medications in new drug applications [22], while the Iyakuhinmei Data File (IDF)s is still used for coding of patient safety data. For companies active in both Japan and other countries, the CRT Japan was created to offer a simple solution for conversion between the IDF and WHODrug Drug codes.

The European Medicines Agency (EMA) requests the most specific ATC, i.e. the fifth level, for submission of medicinal products. Since all medicinal products and ingredients in WHODrug are generally assigned with ATC codes on the fourth level, the CR ATC 5 was created to facilitate the submission process and help translate the Drug code to ATC level five assignments.

### Display Data in Another Language

WHODrug has a global scope using non-proprietary names in English as standard. This is enough for many users, both regulatory authorities and pharmaceutical companies. However, in some cases the medications do not have an English name, non-proprietary or common. In order to facilitate the use of WHODrug in all countries, UMC has started to develop versions of the dictionary that can handle different types of languages, for instance Chinese. The possibility to use Chinese characters enables medication coding in Chinese, for efficient and standardised handling of medicinal data as well as its submission, without the need for manual and time-consuming translations to the English language. The Chinese version also enables automated and instant translations back and forth between English and Chinese medication information, allowing simplified data submissions to authorities both within and outside China.

### Automation for Efficient Coding

The vigorous activity of coding medicinal information is of great importance for patient safety but can be time consuming and challenging. The growing number of medications available on the global market increases the need for automated services for coding of medications. WHODrug Koda combines the use of Artificial Intelligence as well as in-depth and continuous training of the engine based on industry and UMC insights on coding using WHODrug.

## Continuous WHODrug Updates

New medications are continuously released onto the market and therefore WHODrug is updated regularly with new medication names and ATC code assignments. Because registration, coding and analysis of concomitant medications have become increasingly important when handling clinical trial data, updating to the most recent version of WHODrug is essential. Upversioning of WHODrug may simplify analysis by ensuring the most up-to-date coding selections. Using the most recent version of WHODrug is especially important in clinical and observational trials where many new medications are used as concomitant medication (e.g. oncology trials) and in post-marketing safety coding, since they often concern medications that are new on the market.

There are different strategies to handle the releases of updated versions of WHODrug in relation to the coding conducted in earlier versions. By having all trials within a programme or the entire safety database coded to the same version, the most accurate coding can be achieved with lower risk of having a regulatory authority reject the data. WHODrug Change Analysis Tool (CAT) can be used to analyse changes (i.e. modifications, deletions and insertions) between two versions of WHODrug and thereby the impact of the upversioning and the workload involved can be predicted.

## Final Remarks

What started out as an in-house tool for managing medicinal products reported in ICSR’s has grown to be the world’s largest and foremost medicinal dictionary of its kind, with a unique global coverage of medicinal products and ingredients. The pharmaceutical industry is expanding across the globe and new types of treatments that could not be imagined 40 years ago are coming into use. The basic structure of WHODrug has been the same over the years, but the data presentation has developed to support international standardisation efforts and efficient analysis both within the WHO PIDM and for drug development.

## References

[1] Babre D (2010). Medical coding in clinical trials. Perspect Clin Res..

[2] Lu Z (2010). Technical challenges in designing post-marketing eCRFs to address clinical safety and pharmacovigilance needs. Contemp Clin Trials..

[3] Nair GJ (2013). Ensuring quality in the coding process: a key differentiator for the accurate interpretation of safety data. Perspect Clin Res..

[4] FDA. FDA Data Standards Catalog v5.2 (12-20-2018). In. https://www.fda.gov/industry/fda-resources-data-standards/study-data-standards-resources: FDA.

[5] PMDA. Notification on Practical Operations of Electronic Study Data Submissions In: Bureau PaFS, ed. https://www.pmda.go.jp/english/review-services/reviews/0002.html: PMDA; 2019.

[6] Bejan-Angoulvant T, Genet T, Vrignaud L, Angoulvant D, Fauchier L (2018). Three case reports of involuntary muscular movements as adverse reactions to sacubitril/valsartan. Br J Clin Pharmacol.

[7] Juhlin K, Karimi G, Andér M (2015). Using VigiBase to identify substandard medicines: detection capacity and key prerequisites. Drug Saf.

[8] van Stekelenborg J, Ellenius J, Maskell S (2019). Recommendations for the use of social media in pharmacovigilance: lessons from IMI WEB-RADR. Drug Saf.

[9] Ellenius JBT, Dasgupta N, Hedfors S, Pierce C, Norén GN (2016). Medication name entity recognition in tweets using global dictionary lookup and word sense disambiguation. Pharmacoepidemiol Drug Saf.

[10] Star K, Sandberg L, Bergvall T, Choonara I, Caduff-Janosa P, Edwards IR (2019). Paediatric safety signals identified in VigiBase: methods and results from Uppsala Monitoring Centre. Pharmacoepidemiol Drug Saf.

[11] Wakao R, Taavola H, Sandberg L (2019). Data-driven identification of adverse event reporting patterns for Japan in VigiBase, the WHO Global Database of Individual Case Safety Reports. Drug Saf.

[12] Vargesson N (2015). Thalidomide-induced teratogenesis: history and mechanisms. Birth Defects Res Part C.

[13] The WHO Programme for International Drug Monitoring. 2019; https://www.who.int/medicines/areas/quality_safety/safety_efficacy/National_PV_Centres_Map/en/.

[14] Lindquist M (2008). VigiBase, the WHO Global ICSR Database System: basic Facts. Drug Inf J..

[15] Helling M, Venulet J (1974). Drug recording and classification by the WHO research centre for international monitoring of adverse reactions to drugs. Methods Inf Med.

[16] Venulet J, Borda MH (2010). WHO’s international drug monitoring—the formative years, 1968–1975. Drug Saf..

[17] FDA US. 21 e-C.F.R. §314:3, 19 December. In: 2019.

[18] WHO Collaborating Centre for Drug Statistics Methodology, Guidelines for ATC classification and DDD assignment, 2019. Oslo, 2018. In:2019.

[19] Watson S, Härmark L (2018). Desogestrel and panic attacks—a new suspected adverse drug reaction reported by patients and health care professionals on spontaneous reports. Br J Clin Pharmacol.

[20] Heiko Baermann; Matthias Frischmann. Drug Groupings and workflow options for the processing and review of concomitant medication data. *PhUSE Annual Conference—Brussels 14th October.* 2013.

[21] Rebecca E, Chandler OL. The utilisation of a new tool in signal management—WHODrug Standardised Drug Groupings. ICPE; 2019; Philadelphia.

[22] PMDA. Notification on Practical Operations of Electronic Study Data Submissions In: Bureau PaFS, ed. https://www.pmda.go.jp/english/review-services/reviews/advanced-efforts/0002.html: PMDA; 2015.

